# Independent and joint effect of relative telomere length and type 2 diabetes on all-cause mortality in American adults

**DOI:** 10.3389/fendo.2022.1035017

**Published:** 2022-11-10

**Authors:** Beidi Lan, Yuan Bai, Xiaoyi Chang, Xiaoxia Zhang

**Affiliations:** ^1^ Department of Structural Heart Disease, The First Affiliated Hospital of Xi’an Jiaotong University, Xi’an, China; ^2^ Department of Cardiology, Changhai Hospital of The Navy Military Medical University, Shanghai, China; ^3^ Department of State-Owned Assets Management, The First Affiliated Hospital of Xi’an Jiaotong University, Xi’an, China; ^4^ Department of Pharmacy, The First Affiliated Hospital, Xi’an Jiaotong University, Xi’an, Shaanxi, China

**Keywords:** telomere length, diabetes, mortality, NHANES, database

## Abstract

**Objective:**

The joint effect of leukocyte telomere length (LTL) and type 2 diabetes (T2D) on the risk of all-cause death has been sparsely explored. The study designed to examine the joint effect of T2D and LTL on the probability of death in American adults.

**Methods:**

A cohort of 6862 adults with LTL measurements and with or without T2D from the NHANES 1999-2002 with follow-up information until 2015 was studied. Quantitative PCR was used to measure the length of telomeres relative to standard reference DNA (T/S ratio). Individuals were grouped into three tertiles according to the LTL levels, with the first tertile demonstrating the lowest one and used as the reference group. The effects of LTL and T2D status on death were evaluated using Kaplan–Meier curves along with log-rank test. Three Cox proportional hazards models with adjustment for various confounders were used to examine the links between TL and all-cause death possibility using adjusted hazard ratios (HRs).

**Results:**

Adults in the sample averaged 45.54 years of age, with 49.51% being male. After a median follow-up period of 14.4 years, 1543 (22.5%) individuals died from all cause. The probability of all-cause mortality was higher among individuals with LTL in the highest tertile than individuals in the lowest tertile (aHR = 0.89; 95%CI: 0.77-1.03); however, the difference did not reach the level of statistical significance (*P* = 0.11). Conversely, the individuals with T2D had a higher probability of death than individuals without (aHR = 1.26; 95%CI: 1.06-1.50; *P* = 0.0092). When LTL and T2D status were investigated jointly, subjects in the highest TLT tertile and with T2D had the highest probability of mortality compared with their counterparts (aHR = 1.34; 95%CI: 1.07-1.68; *P* = 0.0101). However, there was no independent effect of low TLT on mortality as demonstrated among individuals with diabetes (aHR = 1.14; 95%CI: 0.95-1.38; *P* = 0.1662).

**Conclusion:**

The joint effect of TLT and T2D was larger than the sum of the independent effects on the risk of all-cause death. Participants with high TLT and diabetes showed the highest possibility of death compared with other groups.

## Introduction

The worldwide prevalence of diabetes in adults has increased dramatically over recent decades, which has become as a significant cause of morbidity and mortality globally (1). Type 2 diabetes (T2D) is a chronic metabolic disorder related to multiple complications with a worldwide distribution, which characterized by insulin resistance and hyperglycemia and can affect multiple organ systems (2). Among the numerous chronic diseases, T2D is now one of the most recognized diseases globally and it is anticipated that by the year 2030 around 580 million individuals will suffer from it (3, 4).

The telomeres at the ends of chromosomes serve as protective structures for the ends of eukaryotic chromosomes (5). Telomere shortening is associated with older age as well as undesirable lifestyle factors such as smoking, overweight and alcohol abuse (6). There is emerging evidence that the length of leukocyte telomeres (LTL) can serve as a marker for organismal aging, as well as for well-established age-related diseases such as coronary heart disease, Alzheimer’s disease, and diabetes (7–10). As T2D has been commonly considered as an adult age−related disease, it may be associated with LTL. However, the relationship between LTL and T2D is still not clear. Previous studies have detected telomere shortening in T2D, while others have revealed a negative co−relation of LTL and T2D (11, 12). Furthermore, the significance of LTL as a prognostic indicator for death in the general population is conflicting. Two previous cohort reports have revealed that LTL is a biomarker of mortality (13, 14), while other prospective studies have failed to demonstrate such association (15, 16). As for diabetes, two studies in European populations with relatively small sample size found that LTL was associated with all-cause death in type 1 diabetes, and LTL combined with clinicopathological characteristics can provide additional prognostic significance on death probability in T2D individuals (17, 18). According to a recent study, shorter LTL was associated with an elevated mortality rate among individuals with T2D in the Chinese population (19). Both T2D and LTL were related to aging, however, the effect of exposure to T2D with LTL on all-cause mortality in American adults has not been evaluated till now.

This study designed to explore whether LTL can, independently and jointly with T2D, influence the possibility of all-cause death using data from the NHANES. Understanding this association could contribute to prevent T2D and develop health promotion programs.

## Materials and methods

### Study populations

Information of individuals enrolled in the NHANES 1999–2002, a nationally representative survey sample of civilian noninstitutionalized US population was utilized. In brief, as an ongoing cross-sectional survey, the NHANES surveys using complex, multi-stage and probability sampling approaches to estimate the health and nutritional status of the civilians in US and to provide vital and health statistics for the nation. NHANES organizes the family face-to-face interviews, medical examination, laboratory examination, and further gathers the details about the demographics, lifestyles factors, dietary intake, health status and medical history.

**Figure 1:**
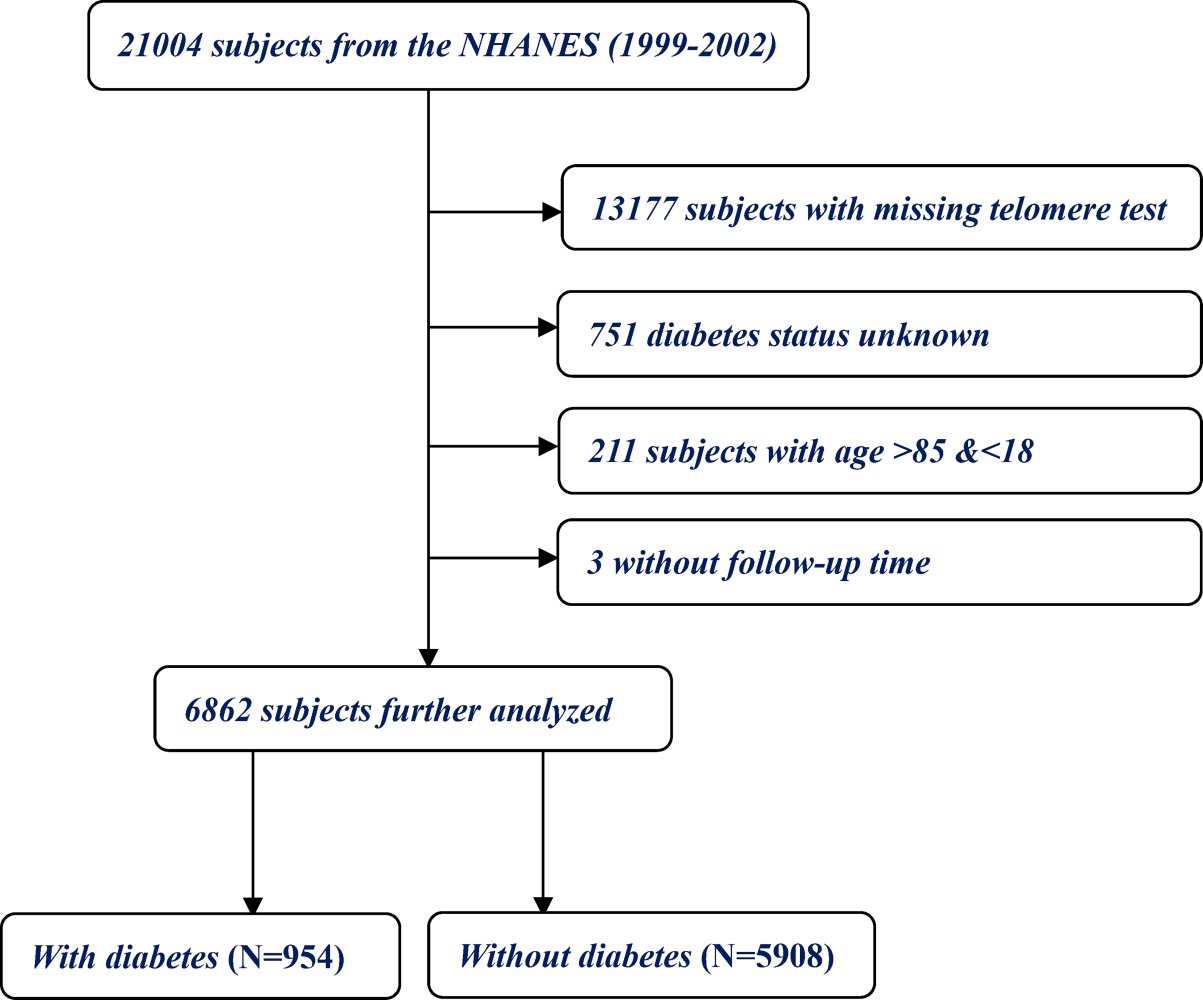
The detailed flow-chart of study population selection.

A total of 21,004 individuals were recorded in the initial analysis. Individuals with missing records for telomere test were excluded (N = 13,177). We further ruled out 751 subjects without reporting diabetes status. Because NHANES recorded who were ≥85 years old as 85 years, subjects younger than 18 years and older than 85 years were excluded (N = 211). Finally, missing data regarding follow-up were excluded (N = 3), thus yielding the sample size to 6,862 eligible adults (**Figure 1**).

### Measurements of LTL

LTL measures are available for two cycles of NHANES from 1999 to 2002. Blood samples containing their DNA were collected from all adults and stored at −80°C at the Centers for Disease Control and Prevention (CDC). For DNA analysis, qPCR assays were carried out at a laboratory in San Francisco, California to measure the telomere length relative to standard reference DNA, as described in detail elsewhere previously (20). LTL was determined and compared as telomere-to-single copy gene ratio (T/S ratio). According to NHANES, qPCR was carried out three times on three different days, and the samples were assayed in duplicate wells to gain 6 data points. Full details regarding the LTL measurement are open and available and listed on the NHANES web at: https://wwwn.cdc.gov/Nchs/Nhanes/2001-2002/TELO_B.htm. The CDC Institutional Review Board provided human subject approval for the study.

### Study variables

The NHANES physical examination in our study mainly involved measurement of waist circumference, height, weight, and body mass index (BMI), which was computed as weight in kg divided by height in m, squared. Information on age (years), sex (male, female), family poverty income ratio, race/ethnicity (Mexican American, non-Hispanic Black, non-Hispanic White, and others), education (less than high school, high school diploma, and more than high school), marital status (married, unmarried), smoking (current smoker, former smoker and never smoker), history of antidiabetic drug, and alcohol drinking was based on self-report during the questionnaire portion of the survey. For simplicity, there were two categories of marital status, namely, unmarried (widowed/divorced/separated/never married) and married (living as married). Alcohol drinking was defined as an individual who had alcohol abuse at least 12 times per year. Furthermore, individuals’ history of cardiovascular disease (CVD), cancer, asthma, and hypertension was obtained. Metabolic Syndrome (MetS) was defined as individuals with the evidence of three or more of five components (abdominal obesity, elevated triglycerides, blood pressure, fasting blood glucose, and reduced high-density lipoprotein cholesterol) (21). Participants were considered to have a history of CVD if they had been told that they had congestive heart failure, angina, stroke, coronary heart disease, or heart attack. According to the American Diabetes Association (22), the diagnostic criteria for T2D are made if any of the following conditions presented (1): doctor told you have diabetes (2); glycohemoglobin HbA1c (%) > 6.5; (3) fasting glucose ≥ 7mol/L; (4) random blood glucose ≥ 11.1 mmol/L; (5) Oral antidiabetic medication. Using the Chronic Kidney Disease Epidemiology Collaboration (CKD-EPI) equation, we estimated the glomerular filtration rate (eGFR) (23). In regard to general biochemistry tests, creatinine (mg/dl), serum albumin (g/dl), C-reactive protein (mg/dl), high-density lipoprotein (HDL) cholesterol (mg/dl), uric acid (mg/dl), and total cholesterol (mg/dl) were enrolled. Details regarding the full procedures of laboratory tests can be obtained on the official website at https://wwwn.cdc.gov/nchs/nhanes.

### Mortality follow-up data

Survival status data was downloaded from the National Death Index (NDI). The NHANES data was merged with the NDI using a unique study identifier. The detailed mortality records are available online at https://www.cdc.gov/nchs/datalinkage/mortality-public.htm. Adult individuals were followed for mortality through December 31, 2015 to ascertain survival status. The primary endpoint event was all-cause death. The cause of death in the present study was based on ICD-10 system codes.

### Statistical analysis

According to the NHANES recommendation and guidelines, an appropriate sampling weight for the variable of interest that was collected on the smallest number of respondents was calculated and accounted for complex multistage survey design strategies in the analysis. In the survey, continuous variables were expressed as a survey-weighted mean with 95% confidence intervals (CI), whereas categorical variables were expressed as survey-weighted percentages with 95% confidence intervals (CI). The LTL levels were divided into three tertiles, with the first tertile representing the lowest level and being considered the reference group. In comparison with those in tertile 1, the hazard ratios (HRs) and 95% CIs for subjects in tertile 2 and 3 were computed. A potential confounder was selected if it altered LTL estimates by more than 10% or was notably associated with mortality (24). The effects of LTL and T2D status on death were evaluated using Kaplan–Meier curves along with log-rank test. Three Cox proportional hazards models were utilized with adjustment for potential confounders. As for model 1, no confounding factors were adjusted. A model 2 had adjustment for age, sex, and smoking. Model 3 was further adjusted for cancer, hypertension, CVD, MetS, eGFR, albumin, and antidiabetic drug. It was considered statistically significant if the *P*-value was less than 0.05. A variety of analyses were performed with Empower software (www.empowerstats.com; X&Y Solutions, Inc., Boston, MA, USA) and R version 3.6.3 (http://www.Rproject.org, The R Foundation).

## Results

### A description of a person’s characteristics

In total, 6862 adults participated in the study, which included 954 individuals with T2D and 5908 without. An overview of the demographic baseline characteristics of the included individuals can be found in **Table 1**. People on average were 45.5 years old, with 49.5% being males. The weighted mean (95% confidence interval) of LTL concentration was 1.06 (1.03, 1.09).

**Table 1:** Baseline characteristics of participants by telomere length tertiles and status of diabetes, weighted.

Characteristics	First to two tertiles (0.39-1.11)	Third tertiles(1.11-3.31)	P-value	Without diabetes	With diabetes	P-value
**Age, years**	49.46 (48.56,50.36)	38.92 (37.65,40.20)	<0.0001	44.26 (43.55,44.98)	57.80 (56.22,59.37)	<0.0001
**Body mass index, kg/m^2^**	28.34 (28.03,28.65)	27.44 (27.05,27.83)	0.0002	27.57 (27.31,27.83)	32.31 (31.43,33.20)	<0.0001
**Waist circumference (cm)**	97.11 (96.31,97.91)	93.50 (92.48,94.51)	<0.0001	94.48 (93.82,95.14)	108.52 (106.47,110.58)	<0.0001
**Family poverty income ratio**	3.05 (2.90,3.21)	2.95 (2.79,3.12)	0.2175	3.06 (2.91,3.20)	2.62 (2.44,2.79)	<0.0001
**Albumin (g/dl)**	4.36 (4.34,4.38)	4.42 (4.38,4.45)	0.0014	4.40 (4.37,4.42)	4.25 (4.21,4.28)	<0.0001
**C-reactive protein(mg/dl)**	0.45 (0.42,0.48)	0.35 (0.31,0.38)	0.0001	0.39 (0.36,0.41)	0.66 (0.60,0.72)	<0.0001
**Creatinine (mg/dl)**	0.85 (0.83,0.87)	0.81 (0.80,0.83)	0.0205	0.83 (0.81,0.84)	0.92 (0.87,0.96)	0.0006
**HDL-cholesterol (mg/dl)**	50.77 (49.72,51.83)	51.37 (50.52,52.22)	0.3547	51.60 (50.75,52.45)	45.18 (44.05,46.30)	<0.0001
**Total cholesterol (mg/dl)**	205.71 (203.26,208.17)	197.67 (195.80,199.53)	<0.0001	202.16 (200.22,204.10)	208.08 (203.06,213.10)	0.0293
**eGFR (CKD-EPI formula), ml/min per 1.73 m^2^**	90.32 (89.38,91.26)	100.24 (99.16,101.33)	<0.0001	95.03 (94.00,96.05)	84.25 (82.35,86.15)	<0.0001
**Uric acid (mg/dl)**	5.41 (5.35,5.47)	5.31 (5.22,5.39)	0.0682	5.34 (5.29,5.39)	5.65 (5.49,5.82)	0.0011
**Sex (%)**			0.5851			0.0016
**Female**	50.82 (49.02,52.62)	49.94 (47.93,51.95)		51.00 (49.89,52.10)	45.67 (42.53,48.84)	
**Male**	49.18 (47.38,50.98)	50.06 (48.05,52.07)		49.00 (47.90,50.11)	54.33 (51.16,57.47)	
**Race (%)**			0.0017			<0.0001
**Mexican American**	6.94 (5.05,9.46)	6.84 (5.11,9.11)		6.89 (5.36,8.83)	7.03 (4.79,10.20)	
**Non-Hispanic Black**	7.69 (5.82,10.10)	11.90 (9.31,15.09)		8.84 (6.98,11.13)	13.34 (10.20,17.27)	
**Non-Hispanic White**	75.89 (71.24,80.00)	68.26 (63.45,72.70)		73.90 (70.14,77.33)	64.86 (58.72,70.55)	
**Others**	9.48 (6.23,14.16)	13.00 (9.15,18.15)		10.37 (7.37,14.42)	14.77 (9.34,22.57)	
**Education (%)**			0.0004			<0.0001
**Less Than High School**	22.51 (20.25,24.93)	18.15 (16.35,20.11)		19.46 (17.61,21.46)	34.55 (30.22,39.16)	
**High School Diploma**	26.48 (24.15,28.94)	25.62 (22.96,28.47)		26.18 (24.00,28.49)	25.91 (22.02,30.23)	
**More Than High School**	51.02 (47.50,54.53)	56.23 (52.91,59.50)		54.35 (51.02,57.65)	39.54 (35.22,44.03)	
**Marital status (%)**			0.0001			0.6852
Unmarried*	32.35 (30.39,34.38)	39.26 (35.86,42.77)		34.74 (32.54,37.02)	35.96 (30.78,41.48)	
**Married**	67.65 (65.62,69.61)	60.74 (57.23,64.14)		65.26 (62.98,67.46)	64.04 (58.52,69.22)	
**Alcohol drinking (%)**			0.1254			<0.0001
**No**	28.11 (24.79,31.68)	24.95 (20.00,30.65)		25.66 (21.93,29.79)	39.24 (35.52,43.08)	
**Yes**	71.89 (68.32,75.21)	75.05 (69.35,80.00)		74.34 (70.21,78.07)	60.76 (56.92,64.48)	
**Smoking (%)**			<0.0001			<0.0001
**Never**	48.12 (45.48,50.77)	53.07 (48.95,57.14)		50.37 (47.71,53.03)	45.96 (41.04,50.96)	
**Former**	28.14 (25.77,30.65)	19.81 (17.71,22.08)		24.10 (22.32,25.98)	34.10 (30.20,38.23)	
**Now**	23.74 (21.53,26.10)	27.13 (23.83,30.69)		25.52 (23.53,27.63)	19.94 (16.88,23.40)	
**Cancer (%)**			<0.0001			<0.0001
**No**	90.74 (89.29,92.00)	95.48 (94.14,96.52)		93.05 (92.01,93.96)	87.21 (83.92,89.90)	
**Yes**	9.26 (8.00,10.71)	4.52 (3.48,5.86)		6.95 (6.04,7.99)	12.79 (10.10,16.08)	
**Hypertension (%)**			<0.0001			<0.0001
**No**	69.30 (66.70,71.77)	79.66 (77.16,81.96)		76.21 (74.02,78.26)	43.79 (39.38,48.29)	
**Yes**	30.70 (28.23,33.30)	20.34 (18.04,22.84)		23.79 (21.74,25.98)	56.21 (51.71,60.62)	
**Asthma (%)**			0.5392			0.935
**No**	88.32 (86.69,89.78)	88.97 (86.95,90.70)		88.55 (87.14,89.82)	88.70 (84.38,91.94)	
**Yes**	11.68 (10.22,13.31)	11.03 (9.30,13.05)		11.45 (10.18,12.86)	11.30 (8.06,15.62)	
**CVD (%)**			<0.0001			<0.0001
**No**	89.64 (88.42,90.74)	96.14 (94.78,97.16)		93.63 (92.75,94.42)	76.89 (72.14,81.04)	
**Yes**	10.36 (9.26,11.58)	3.86 (2.84,5.22)		6.37 (5.58,7.25)	23.11 (18.96,27.86)	
**MetS (%)**			0.0005			<0.0001
**No**	89.96 (88.87,90.97)	93.22 (91.60,94.54)		94.52 (93.74,95.20)	59.01 (54.55,63.32)	
**Yes**	10.04 (9.03,11.13)	6.78 (5.46,8.40)		5.48 (4.80,6.26)	40.99 (36.68,45.45)	
**Antidiabetic**						<0.0001
** No**	93.64 (92.66,94.50)	96.17 (94.77,97.20)	0.0018	100.0(100.0, 100.0)	42.32 (37.66,47.11)	
** Yes**	6.36 (5.50,7.34)	3.83 (2.80,5.23)		0.0(0.0, 0.0)	57.68 (52.89,62.34)	

^*^Included divorced/never married/separated/widowed.

Compared with individuals with LTL in the lower tertile (T1–T2), individuals in the highest tertile (T3) had a lower mean age, BMI, waist circumference, C-reactive protein, creatinine, total cholesterol, high school diploma, married status, history of cancer, hypertension, CVD, and MetS at baseline, and had a higher albumin, eGFR, and now smoking. Compared with individuals without T2D, individuals with T2D had higher mean age, BMI, waist circumference, C-reactive protein, creatinine, total cholesterol, uric acid, and more male, Mexican American, non-Hispanic Black, former smoking, history of cancer, hypertension, CVD, and MetS at baseline. Participants with T2D had lower family poverty income ratio, albumin, HDL-cholesterol, eGFR, alcohol drinking, never smoking, and now smoking.

### Association between LTL, T2D and mortality

During a mean of 160 months of follow-up, 1543 deaths from all causes were identified. In **Table 2**, Cox regression analysis was adopted to assess the individual effect of LTL and T2D on all-cause risk. When LTL was used as continuous variable, LTL was associated with reduced risk of death in the crude model (HR = 0.10, 95%CI= 0.08-0.12; *P* < 0.0001), and model I (aHR = 0.78, 95%CI = 0.62-0.99; *P* = 0.0389). However, in the full adjusted model, LTL was not associated with a lower risk of death (aHR = 0.82, 95%CI= 0.65-0.1.04; *P* = 0.0944). The results remained when LTL was used as categorical variable. LTL in the higher tertiles (T2, T3) was not associated with decreased risk of death in the full adjusted model (aHR = 0.95, 95%CI = 0.85-1.07; *P* = 0.4207; aHR = 0.89, 95%CI = 0.77-1.03; *P* = 0.1100, respectively). When individuals without T2D were used as reference, it was found a significant association between LTL and all-cause mortality in the crude model (HR = 2.95, 95%CI= 2.64-3.29; *P* < 0.0001), model I (aHR = 1.57, 95%CI = 1.40-1.75; *P* < 0.0001), and the full adjusted model (aHR = 1.26, 95%CI = 1.06-1.50; *P* = 0.0092).

**Table 2:** Individual effect of telomere length (T/S ratio) and diabetes on all-cause mortality.

Exposure	Non-adjusted	Adjust I	Adjust II
**Telomere Length (T/S ratio)**	0.10 (0.08, 0.12) <0.0001	0.78 (0.62, 0.99) 0.0389	0.82 (0.65, 1.04) 0.0944
**Telomere Length (T/S ratio) tertiles**			
** Tertile 1**	1 (Ref)	1 (Ref)	1 (Ref)
** Tertile 2**	0.51 (0.46, 0.58) <0.0001	0.91 (0.81, 1.02) 0.1172	0.95 (0.85, 1.07) 0.4207
** Tertile 3**	0.30 (0.26, 0.34) <0.0001	0.86 (0.75, 0.99) 0.0400	0.89 (0.77, 1.03) 0.1100
**Diabetes**			
**No**	1 (Ref)	1 (Ref)	1 (Ref)
**Yes**	2.95 (2.64, 3.29) <0.0001	1.57 (1.40, 1.75) <0.0001	1.26 (1.06, 1.50) 0.0092

Non-adjusted model adjust for: None.

Adjust I model adjust for: Age, sex, and smoking.

Adjust II model adjust for: Age, sex, smoking, cancer, hypertension, CVD, MetS, eGFR, albumin, and antidiabetic drug.

**Figure 2:**
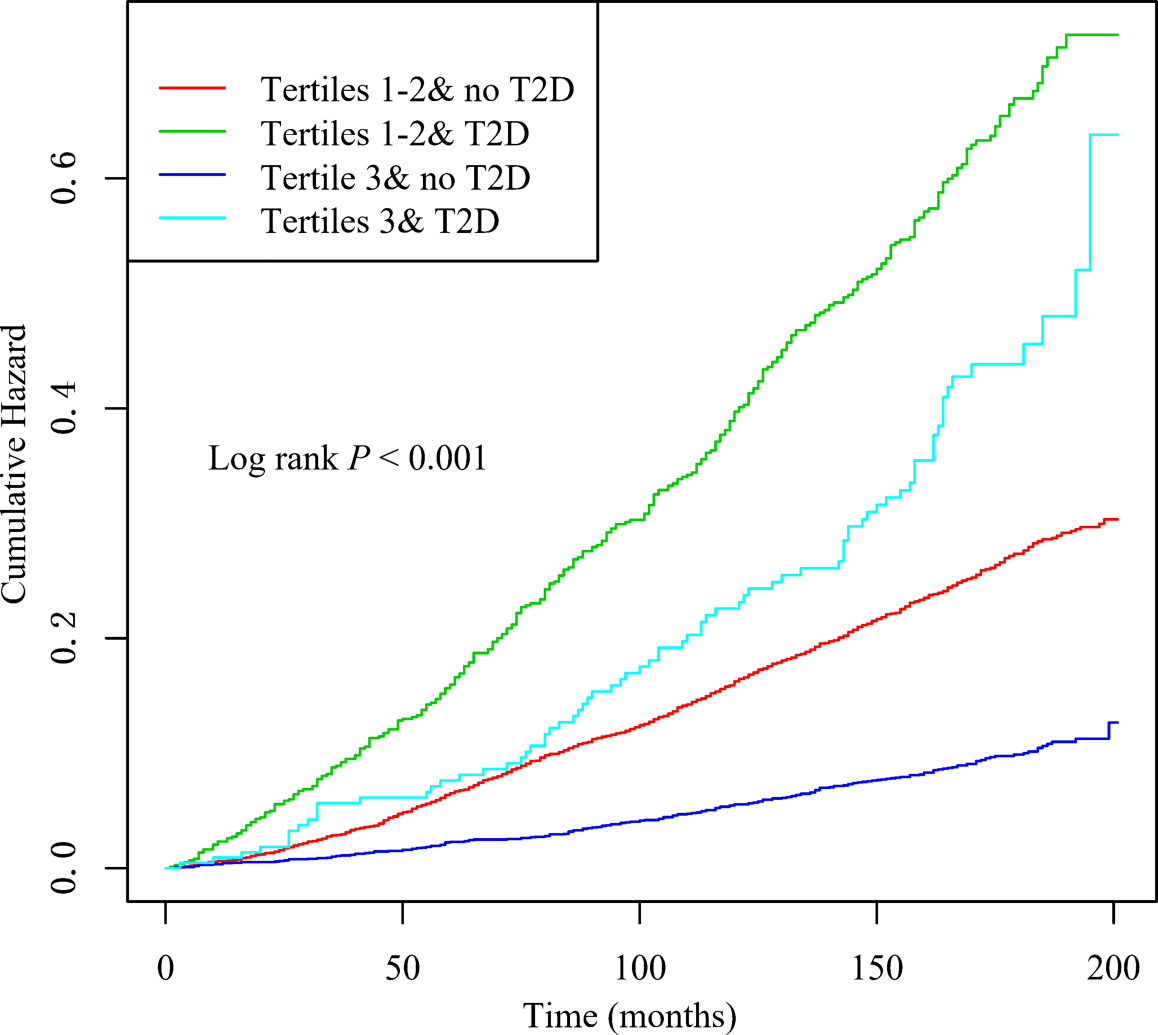
Kaplan–Meier curves of cumulative hazards of all-cause death by baseline leukocyte telomere length (T1–T2 vs T3) and type 2 diabetes status (with, without).

**Figure 3:**
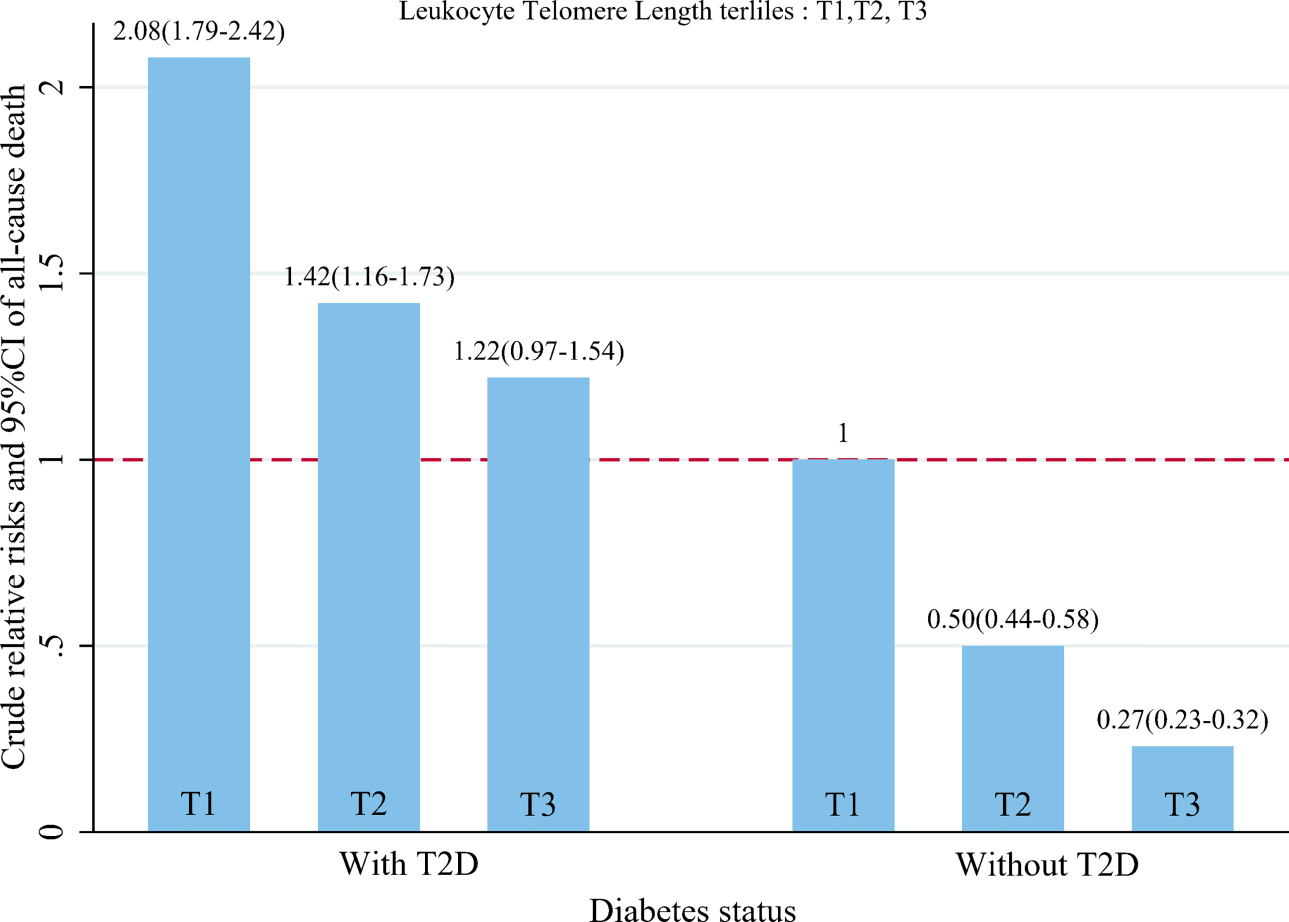
Dose–response relationship between baseline leukocyte telomere length quartiles and crude hazard ratios and 95%CI of all-cause death, stratified by status of T2D.

When LTL and T2D were investigated together, the Kaplan-Meyer curves demonstrated that the cumulative hazard of death notably differed among the four groups defined by LTL and T2D categories (comparing LTL in the T1-T2 and T3 among those individuals without T2D, the *P* < 0.001; comparing LTL in the T1-T2 and T3 among those individuals with T2D, the *P* < 0.0001; **Figure 2**). As presented in **Figure 3**, within each stratum of diabetes, there was a negative association between tertiles of LTL and risk of death in unadjusted model (*P*_for trend_ < 0.001). However, the all-cause death risk was higher among the individuals with T2D.

The Cox proportional hazards regression analyses in **Table 3** revealed that the highest probability of all-cause death was in the group with highest LTL tertile and T2D, with an HR of 1.34 (95% CI = 1.07–1.68; *P* = 0.0101) after adjusting for age, smoking, cancer, sex, CVD, MetS, hypertension, eGFR, albumin, and antidiabetic drug. However, for individuals in lower LTL tertile (T1-T2), the all-cause mortality was comparable regardless of the T2D status (aHR = 1.14, 95% CI = 0.95–1.38; *P* = 0.1662). Nevertheless, in individuals without T2D, higher LTL tertile was associated with a decreased risk of all-cause death when compared with those individuals with lower LTL tertile (aHR = 0.86, 95% CI = 0.75–0.98; *P* = 0.0240).

**Table 3:** Joint effects of baseline telomere length (Tertile 1-2 vs Tertile 3) and status of diabetes (Yes, No) on all-cause mortality.

Combined Groups		Non-adjusted	Adjust I	Adjust II
**DM**	telomere length (T/S ratio)			
**No**	Tertile 1-2	1 (Ref)	1 (Ref)	1 (Ref)
**No**	Tertile 3	0.37 (0.33, 0.42) <0.0001	0.85 (0.74, 0.97) 0.0178	0.86 (0.75, 0.98) 0.0240
**Yes**	Tertile 1-2	2.18 (1.91, 2.50) <0.0001	1.44 (1.26, 1.65) <0.0001	1.14 (0.95, 1.38) 0.1662
**Yes**	Tertile 3	1.57 (1.31, 1.89) <0.0001	1.63 (1.36, 1.96) <0.0001	1.34 (1.07, 1.68) 0.0101

Non-adjusted model adjust for: None.

Adjust I model adjust for: Age, sex, and smoking.

Adjust II model adjust for: Age, sex, smoking, cancer, hypertension, CVD, MetS, eGFR, albumin, and antidiabetic drug.

## Discussion

T2D is characterized by increased blood glucose values, while the LTL is used as a biological biomarker of cell ageing. Previous studies have demonstrated that shortened LTL was significantly associated with incident T2D (11, 25). Thus, this study designed to find the joint effect of T2D and LTL on the risk of mortality in American adults. In this prospective cohort study, the results indicate that regarding all-cause death the joint effect of T2D and elevated LTL is larger than the sum of their individual effects. These results demonstrate that controlling T2D may bring an additional risk reduction regarding all-cause death.

Telomere shortening is confirmed as a key molecular mechanism of vascular aging (26), and shorter telomere length has been related to age-related diseases including T2D (27). Telomere length shortens gradually during each cell division cycle, and is inversely associated with the total times of cell divisions (28). Previous studies have exhibited an association of mean LTL shortening in patients with T2D (29, 30). The study has contributed new insights to LTL and T2D. So far, the role of T2D and LTL on the risk of mortality has not been examined thoroughly.

In our study, we demonstrated a joint effect of T2D and LTL on the risk of mortality in American adults. The highest probability of all-cause death was noted in the group with highest LTL tertile and T2D. Furthermore, we also found that in individuals without T2D, higher LTL tertile was associated with a decreased risk of all-cause death when compared with those individuals with lower LTL tertile. A recent meta-analysis contained 6,991 individuals and 2,011 incident T2D events concluded that the combined relative risk for T2D incidence was 1.31 when comparing the lowest with the highest LTL at baseline (31). These findings support the view that telomere shortening is a pivotal hallmark of cellular senescence and organismal aging. Individuals with shorter LTL at baseline exhibit a higher probability of mortality during follow-up when compared to those with longer LTL in general population after adjusting for multiple traditional risk factors (19). However, not all studies supported a positive link between shorter telomeres and elevated probability of mortality in general population in previous studies that focused on older patients (27, 32). The possible explanation is that telomere shortening has telomeres have ahead presented among elderly individuals. Besides, considering the influence of other potential risk factors for mortality (such as myocardial infarction, malignancy), the independent effect of LTL on all-cause death becomes less significant. Glucose-induced oxidative stress and proinflammatory conditions may be involved in T2D-induced LTL shortening (33). It has confirmed that inflammation contributes to the loss of telomere length in cultured proliferative cells. Therefore, theoretically, telomere length is impacted by the number of replications and inflammation (34). Furthermore, in animal studies, it has established that hyperglycemia reduce endothelial cell nitric oxide production, contributed to inflammation and oxidative stress, and stimulated LTL shortening and vascular atherosclerotic processes (35, 36). In addition, oxidative stress is elevated both in leukocytes and pancreatic β-cells, which may lead to LTL aberrantly shortening of β-cell and dysfunction during progressive cell divisions in insulin secretion (37). Those persons with T2D are particularly prone to obesity, hyperglycemia, and chronic inflammation, which exacerbates the LTL shortening process while contributing to atherogenesis (38). Telomere shortening was related to T2D complications, such as diabetic nephropathy, and microalbuminuria, while telomere shortening appears to be weakened in individuals with fairly well controlled T2D (39). These studies showed that oxidative stress may contribute to the decline of telomeres and the development of T2D. These findings further support the results that regarding all-cause death the joint effect of T2D and elevated LTL is larger than the sum of their individual effects.

In this prospective cohort study, a nationally representative survey of US adults is used to examine the joint effect of T2D and long-term care on mortality from all causes. However, there are several shortcomings in this study that need to be addressed. Firstly, it is possible that the measurement of LTL at baseline does not reveal all behavioral changes during follow-up. Furthermore, although multiple factors were adjusted in different models, unmeasured confounders associated with T2D and LTL may influence the findings.

## Conclusion

In a large survey conducted among US adults, it was found that the joint effect of TLT and T2D was larger than the sum of the independent effects on the risk of all-cause death. Participants with high TLT and diabetes had the highest risk of death compared with other groups.

## Data availability statement

Publicly available datasets were analyzed in this study. This data can be found here: Publicly National Health and Nutrition Examination Survey dataset was analyzed in this study. They are publicly available at https://www.cdc.gov/nchs/nhanes/index.

## Ethics statement

Ethical review and approval was not required for the study of human participants in accordance with the local legislation and institutional requirements. Written informed consent from the patients OR patients legal guardian/next of kin was not required to participate in this study in accordance with the national legislation and the institutional requirements.

## Author contributions

BL and XZ contributed to the conception or design of the work. BL and YB contributed to the acquisition, analysis, or interpretation of data for the work. BL, YB, and XC drafted the manuscript. XZ critically revised the manuscript. All authors contributed to the article and approved the submitted version.

## Conflict of interest

The authors declare that the research was conducted in the absence of any commercial or financial relationships that could be construed as a potential conflict of interest.

## Publisher’s note

All claims expressed in this article are solely those of the authors and do not necessarily represent those of their affiliated organizations, or those of the publisher, the editors and the reviewers. Any product that may be evaluated in this article, or claim that may be made by its manufacturer, is not guaranteed or endorsed by the publisher.

## References

[1] Guariguata L Whiting DR Hambleton I Beagley J Linnenkamp U Shaw JE. Global estimates of diabetes prevalence for 2013 and projections for 2035. Diabetes Res Clin Pract (2014) 103(2):137–49. doi: 10.1016/j.diabres.2013.11.002 24630390

[2] Papatheodorou K Banach M Bekiari E Rizzo M Edmonds M. Complications of diabetes 2017. J Diabetes Res (2018) 2018:3086167. doi: 10.1155/2018/3086167 29713648 PMC5866895

[3] Saeedi P Petersohn I Salpea P Malanda B Karuranga S Unwin N. Global and regional diabetes prevalence estimates for 2019 and projections for 2030 and 2045: Results from the international diabetes federation diabetes atlas, 9(th) edition. Diabetes Res Clin Pract (2019) 157:107843. doi: 10.1016/j.diabres.2019.107843 31518657

[4] Cheng F Carroll L Joglekar MV Januszewski AS Wong KK Hardikar AA. Diabetes, metabolic disease, and telomere length. Lancet Diabetes Endocrinol (2021) 9(2):117–26. doi: 10.1016/s2213-8587(20)30365-x 33248477

[5] Blackburn EH. Telomere states and cell fates. Nature (2000) 408(6808):53–6. doi: 10.1038/35040500 11081503

[6] Turner KJ Vasu V Griffin DK. Telomere biology and human phenotype. Cells (2019) 8(1). doi: 10.3390/cells8010073 PMC6356320 30669451

[7] Jenkins EC Marchi EJ Velinov MT Ye L Krinsky-McHale SJ Zigman WB. Longitudinal telomere shortening and early alzheimer's disease progression in adults with down syndrome. Am J Med Genet B Neuropsychiatr Genet (2017) 174(8):772–8. doi: 10.1002/ajmg.b.32575 28856789

[8] Ding H Chen C Shaffer JR Liu L Xu Y Wang X. Telomere length and risk of stroke in Chinese. Stroke (2012) 43(3):658–63. doi: 10.1161/strokeaha.111.637207 22343648

[9] Brouilette SW Moore JS McMahon AD Thompson JR Ford I Shepherd J. Telomere length, risk of coronary heart disease, and statin treatment in the West of Scotland primary prevention study: a nested case-control study. Lancet (2007) 369(9556):107–14. doi: 10.1016/s0140-6736(07)60071-3 17223473

[10] Fyhrquist F Tiitu A Saijonmaa O Forsblom C Groop PH. Telomere length and progression of diabetic nephropathy in patients with type 1 diabetes. J Intern Med (2010) 267(3):278–86. doi: 10.1111/j.1365-2796.2009.02139.x 19563389

[11] Zhao J Miao K Wang H Ding H Wang DW. Association between telomere length and type 2 diabetes mellitus: a meta-analysis. PloS One (2013) 8(11):e79993. doi: 10.1371/journal.pone.0079993 24278229 PMC3836967

[12] Asiimwe D Mauti GO Kiconco R. Prevalence and risk factors associated with type 2 diabetes in elderly patients aged 45-80 years at kanungu district. J Diabetes res 2020 (2020). doi: 10.1155/2020/5152146

[13] Deelen J Beekman M Codd V Trompet S Broer L Hägg S. Leukocyte telomere length associates with prospective mortality independent of immune-related parameters and known genetic markers. Int J Epidemiol (2014) 43(3):878–86. doi: 10.1093/ije/dyt267 PMC4052133 24425829

[14] Rode L Nordestgaard BG Bojesen SE. Peripheral blood leukocyte telomere length and mortality among 64,637 individuals from the general population. J Natl Cancer Inst 107(6) djv074 (2015). doi: 10.1093/jnci/djv074 25862531

[15] Svensson J Karlsson MK Ljunggren Ö. Tivesten Å. Mellström D Movérare-Skrtic S. Leukocyte telomere length is not associated with mortality in older men. Exp Gerontol (2014) 57:6–12. doi: 10.1016/j.exger.2014.04.013 24793325

[16] Houben JM Giltay EJ Rius-Ottenheim N Hageman GJ Kromhout D. Telomere length and mortality in elderly men: the zutphen elderly study. J Gerontol A Biol Sci Med Sci (2011) 66(1):38–44. doi: 10.1093/gerona/glq164 20889650

[17] Astrup AS Tarnow L Jorsal A Lajer M Nzietchueng R Benetos A. Telomere length predicts all-cause mortality in patients with type 1 diabetes. Diabetologia (2010) 53(1):45–8. doi: 10.1007/s00125-009-1542-1 19802713

[18] Bonfigli AR Spazzafumo L Prattichizzo F Bonafè M Mensà E Micolucci L. Leukocyte telomere length and mortality risk in patients with type 2 diabetes. Oncotarget (2016) 7(32):50835–44. doi: 10.18632/oncotarget.10615 PMC5239440 27437767

[19] Cheng F Luk AO Wu H Lim CKP Carroll L Tam CHT. Shortened relative leukocyte telomere length is associated with all-cause mortality in type 2 diabetes- analysis from the Hong Kong diabetes register. Diabetes Res Clin Pract (2021) 173:108649. doi: 10.1016/j.diabres.2021.108649 33422583

[20] Needham BL Adler N Gregorich S Rehkopf D Lin J Blackburn EH. Socioeconomic status, health behavior, and leukocyte telomere length in the national health and nutrition examination survey, 1999-2002. Soc Sci Med (2013) 85:1–8. doi: 10.1016/j.socscimed.2013.02.023 23540359 PMC3666871

[21] Grundy SM Cleeman JI Daniels SR Donato KA Eckel RH Franklin BA. Diagnosis and management of the metabolic syndrome: an American heart Association/National heart, lung, and blood institute scientific statement. Circulation (2005) 112(17):2735–52. doi: 10.1161/circulationaha.105.169404 16157765

[22] American Diabetes Association. 2. classification and diagnosis of diabetes: Standards of medical care in diabetes-2020. Diabetes Care (2020) 43(Suppl 1):S14–s31. doi: 10.2337/dc20-S002 31862745

[23] Levey AS Stevens LA Schmid CH Zhang YL Castro AF Feldman HI. Coresh: A new equation to estimate glomerular filtration rate. Ann Intern Med (2009) 150(9):604–12. doi: 10.7326/0003-4819-150-9-200905050-00006 PMC2763564 19414839

[24] Jaddoe VW de Jonge LL Hofman A Franco OH Steegers EA Gaillard R. First trimester fetal growth restriction and cardiovascular risk factors in school age children: population based cohort study. Bmj 348 g14 (2014). doi: 10.1136/bmj.g14 PMC3901421 24458585

[25] Wang J Dong X Cao L Sun Y Qiu Y Zhang Y. Association between telomere length and diabetes mellitus: A meta-analysis. J Int Med Res (2016) 44(6):1156–73. doi: 10.1177/0300060516667132 PMC5536737 28322101

[26] Serrano AL Andrés V. Telomeres and cardiovascular disease: does size matter? Circ Res (2004) 94(5):575–84. doi: 10.1161/01.Res.0000122141.18795.9c 15031270

[27] Fitzpatrick AL Kronmal RA Gardner JP Psaty BM Jenny NS Tracy RP. Leukocyte telomere length and cardiovascular disease in the cardiovascular health study. Am J Epidemiol (2007) 165(1):14–21. doi: 10.1093/aje/kwj346 17043079

[28] Simm A Nass N Bartling B Hofmann B Silber RE Navarrete Santos A. Potential biomarkers of ageing. Biol Chem (2008) 389(3):257–65. doi: 10.1515/bc.2008.034 18208349

[29] Zee RY Castonguay AJ Barton NS Germer S Martin M. Mean leukocyte telomere length shortening and type 2 diabetes mellitus: a case-control study. Transl Res (2010) 155(4):166–9. doi: 10.1016/j.trsl.2009.09.012 20303464

[30] Masi S D'Aiuto F Cooper J Salpea K Stephens JW Hurel SJ. Telomere length, antioxidant status and incidence of ischaemic heart disease in type 2 diabetes. Int J Cardiol (2016) 216:159–64. doi: 10.1016/j.ijcard.2016.04.130 PMC4900130 27156058

[31] Willeit P Raschenberger J Heydon EE Tsimikas S Haun M Mayr A. Leucocyte telomere length and risk of type 2 diabetes mellitus: new prospective cohort study and literature-based meta-analysis. PloS One (2014) 9(11):e112483. doi: 10.1371/journal.pone.0112483 25390655 PMC4229188

[32] Harris SE Deary IJ MacIntyre A Lamb KJ Radhakrishnan K Starr JM. The association between telomere length, physical health, cognitive ageing, and mortality in non-demented older people. Neurosci Lett (2006) 406(3):260–4. doi: 10.1016/j.neulet.2006.07.055 16919874

[33] Tamura Y Takubo K Aida J Araki A Ito H. Telomere attrition and diabetes mellitus. Geriatr Gerontol Int (2016) 16 Suppl:1, 66–74. doi: 10.1111/ggi.12738 27018285

[34] von Zglinicki T. Role of oxidative stress in telomere length regulation and replicative senescence. Ann N Y Acad Sci (2000) 908:99–110. doi: 10.1111/j.1749-6632.2000.tb06639.x 10911951

[35] Rojas A Romay S González D Herrera B Delgado R Otero K. Regulation of endothelial nitric oxide synthase expression by albumin-derived advanced glycosylation end products. Circ Res (2000) 86(3):E50–4. doi: 10.1161/01.res.86.3.e50 10679490

[36] Schmidt AM Hori O Brett J Yan SD Wautier JL Stern D. Cellular receptors for advanced glycation end products. implications for induction of oxidant stress and cellular dysfunction in the pathogenesis of vascular lesions. Arterioscler Thromb (1994) 14(10):1521–8. doi: 10.1161/01.atv.14.10.1521 7918300

[37] Ihara Y Toyokuni S Uchida K Odaka H Tanaka T Ikeda H. Hyperglycemia causes oxidative stress in pancreatic beta-cells of GK rats, a model of type 2 diabetes. Diabetes (1999) 48(4):927–32. doi: 10.2337/diabetes.48.4.927 10102716

[38] Salpea KD Talmud PJ Cooper JA Maubaret CG Stephens JW Abelak K. Association of telomere length with type 2 diabetes, oxidative stress and UCP2 gene variation. Atherosclerosis (2010) 209(1):42–50. doi: 10.1016/j.atherosclerosis.2009.09.070 19889414 PMC2839074

[39] Salpea KD Humphries SE. Telomere length in atherosclerosis and diabetes. Atherosclerosis (2010) 209(1):35–8. doi: 10.1016/j.atherosclerosis.2009.12.021 PMC2862289 20080237

